# Angiopoietin-like 2 is a potential biomarker for diabetic foot patients

**DOI:** 10.1186/s12902-020-00657-7

**Published:** 2020-11-30

**Authors:** Yan Wang, Zhaohui Zheng, Yuxian Yang, Jianan Lang, Ning Zhang, Longyan Yang, Dong Zhao

**Affiliations:** 1 Center for Endocrine Metabolism and Immune Diseases, Beijing Luhe Hospital, Capital Medical University, Beijing, 101149 China; 2 Beijing Key Laboratory of Diabetes Research and Care, Beijing, 101149 China

**Keywords:** Angiopoietin-like 2, Type 2 diabetes, Diabetic foot ulcers, Biomarker

## Abstract

**Background:**

Circulating angiopoietin-like 2 **(**ANGPTL2) protein levels are known to be significantly increased in numerous chronic inflammatory diseases and are associated with the diagnosis and/or prognosis of cardiovascular diseases, diabetes, chronic kidney disease, and various types of cancers. However, no data regarding the relationship between ANGPTL2 and diabetic foot ulcers (DFUs) are available. Here, we explored the potential link between ANGPTL2 and DFUs.

**Methods:**

A total of 68 participants with type 2 diabetes mellitus (T2DM) were recruited, including 28 patients with DFU and 40 diabetic patients without DFUs. The clinical characteristics of T2DM patients with and without DFUs were compared. Serum concentrations of ANGPTL2 and VEGF were measured using enzyme-linked immunosorbent assay (ELISA) kits. The correlations between ANGPTL2 and clinical variables were analyzed**.** Multiple linear regression and logistic regression models were constructed to test the associations between ANGPTL2 and the severity and presence of DFUs.

**Results:**

Serum levels of ANGPTL2 were higher in patients with DFUs than those in diabetic controls. Serum ANGPTL2 levels were higher in the advanced stages of DFUs. Spearman correlation analysis revealed strong positive associations of ANGPTL2 with CRP, VEGF and ESR in all subjects. In addition, serum ANGPTL2 was still positively correlated with DFUs stage after adjusting the risk factors. After adjusting for age, sex, HbA1C and duration of diabetes, ANGPTL2 was found to be independently associated with the presence of DFUs.

**Conclusions:**

Circulating ANGPTL2 levels are an independent risk factor for DFUs. This suggests that ANGPTL2 may play important roles in the development of DFUs, a possibility that needs to investigated in prospective studies.

## Background

Type 2 diabetes mellitus (T2DM), a chronic inflammatory disease, is widely considered to be a major health issue globally. Long-term disturbance of glycol-metabolism can lead to macrovascular, microvascular and neuropathic diseases, which are the main cause of death and disability in diabetic patients [1]. Diabetic foot ulcers (DFUs) are one of the common complications in T2DM patients and are chartered by lower extremity vascular obstructions, persistent foot infections, ulcers, and deep tissue destruction [2, 3]. They are reported in 15 to 25% of diabetic patients [4, 5]. Currently, comprehensive methods, including debridement, infection control, blood glucose regulation, and vascular opening, are commonly used to treat DFUs [6]. The cost of treatment is close to the sum of the cost of other diabetic complications [7]. However, DFUs do not heal well, which eventually leads to amputation or death. Therefore, it is very important to find a potential biomarker and new therapeutic target for DFUs.

Angiopoietin-like 2 (ANGPTL2, also named ARP2; HARP) is a 64-kDa glycosylated protein belonging to the angiopoietin-like family. It contains a coiled-coil domain and a fibrinogen-like domain that are similar in the structure to angiopoietin [8]. ANGPTL2 is expressed in multiple tissues and organs and is detected in the systemic circulation [9, 10]. Increasing evidence has suggested that excess ANGPTL2 signaling leads to chronic inflammation, resulting in metabolic diseases [10]. ANGPTL2 increases tissue inflammation and tissue reconstruction to maintain tissue homeostasis through integrin α5β1/MAPKs and NF-κB signaling [9, 10]. Moreover, ANGPTL2 promotes adiposity, chronic adipose tissue inflammation, and systemic insulin resistance [11]. Circulating ANGPTL2 level has been shown to be associated with various chronic inflammatory diseases, including T2DM [12], with significantly higher serum levels present in these diseases. The serum ANGPTL2 concentration was reported to be independently associated with death and major adverse cardiovascular events in patients with T2DM [13]. El-Lebedy D. et al. further confirmed that serum ANGPTL2 is an independent risk biomarker for cardiovascular disease in T2DM patients [14]. Moreover, observational clinical studies reported that circulating ANGPTL2 is a risk factor for lower extremity amputation and lower extremity artery disease in a large cohort of T2DM patients [15, 16]. However, no data regarding the relationship between ANGPTL2 levels and DFUs are available. Hence, the present study aimed to explore the association between ANGPTL2 and DFUs.

## Methods

### Subjects

Sixty-eight patients with diagnosed with T2DM were recruited from Beijing Luhe Hospital, Capital Medical University, from January 2016 to October 2017. The study complied with the Helsinki Declaration for the investigation of human subjects. The Ethics Committee of Beijing Luhe Hospital approved this study. Informed consent was obtained from each participatant enrolled in the study. The subjects were divided into two groups: 28 patients with DFUs (DFU group) and 40 patients without DFUs (control group).

The diagnosis of T2DM was based on the 1999 World Health Organization Criteria, which fulfills random blood glucose ≥11.1 mmol/L and/or fasting blood glucose (FBG) ≥ 7.0 mmol/L and/or 2 h blood glucose, during an oral glucose tolerance test (OGTT) ≥ 11.1 mmol/L. Diabetic patients with DFUs were staged according to the Wagner classification system, and DFU was defined as a full-thickness skin break at least to Wagner stage I [17].

All age of the subjects ranged from 20 years to 80 years old. Duration of diabetes, smoking, alcohol consumption, and history of diabetic, complications, were recorded. Patients with other types of diabetes, chronic organ failure, cerebrovascular diseases or other chronic disorders, or who were pregnant, or within 1 year of postpartum period, as well as those with infectious diseases, were excluded.

### Clinical measurements

Venous blood samples were withdrawn from each patient in the morning after overnight fasting. Sera were separated within 1 h. Routine laboratory measurements were performed, including fasting plasma glucose (FBG, mmol/L), alanine transaminase (ALT,U/L), aspartate transaminase (AST,U/L), glutamyl transpeptidase (γ-GT,U/L), triglyceride (TG, mmol/L), total cholesterol (CHO, mmol/L), low density lipoprotein cholesterol (LDL-C, mmol/L), high density lipoprotein cholesterol (HDL-C, mmol/L), C-reactive protein (CRP), erythrocyte sedimentation rate (ESR), and white blood cell count (WBC). Serum samples were stored immediately at − 80 °C until further analysis.

### Assessment of circulating ANGPTL2 and VEGF

Serum ANGPTL2 (1F-716, Immuno-biological laboratories Co., Ltd., Japan) and VEGF (MMV00, R&D Systems, Minneapolis, MN, USA) levels were measured by using a commercially available human ELISA kits according to the manufacturers’ instructions, respectively.

### Statistical analysis

Statistical analyses were performed using the SPSS 18.0 software (SPSS Inc., Chicago, IL, USA). Continuous variables were expressed as the mean ± standard deviation (SD) or median (interquartile ranges) for values with a skewed distribution, such as CRP, ESR and VEGF. The differences between two groups were analyzed by the Student’s t-test or Mann-Whitney test. For comparisons among multiple groups, one-way ANOVA followed by Tukey’s post-hoc analysis were conducted. Spearman correlation analysis was used to examine the correlations between serum ANGPTL2 levels and CRP levels, ESR levels, VEGF levels and DFU stages. Pearson correlation analysis was conducted to examine the correlation between serum ANGPTL2 levels and the clinical and biochemical variables. Multiple linear regression was used to evaluate the multivariate relationships. The stepwise multiple regression model was constructed based on all subjects, considering ANGPTL2 levels as response variables, and including HDL-C, monocytes, lymphocytes and DFU stage, which were regarded as explanatory variables. Collinearity was assessed by calculating the variance inflation factor (VIF), and variables with VIF ≥ 5 were excluded from the models. Binary logistic regression analysis was conducted to detect the independent effect of ANGPTL2 on the presence of DFU, considering DFU occurrences as response variables, and sex, age, T2DM course, HbA1c, and ANGPTL2 levels as covariates. *P* values < 0.05 were considered statistically significant.

## Results

### Clinical characteristics of the study subjects

The characteristics of the study subjects according to the two groups are shown in Table 1. Patients in the two groups were comparable in age, sex, age, diabetes duration, blood pressure, glycemic markers, lipid profiles, and indicators of liver function. The DFU group had significantly higher values of WBC, lymphocytes, CRP and ESR than the control group.

**Table 1:** Clinical and biochemical characteristics of participants

Variables	Diabetic control (*n* = 40)	DFU (*n* = 28)	*P* value
Age (year)	63.55 ± 2.22	65.32 ± 2.65	0.6101
Sex (Female,%)	16 (40.0%)	13 (46.4%)	0.5981
Drinking(n,%)	6 (15.0%)	4 (14.3%)	0.0008*
Diabetes duration (year)	11.99 ± 1.47	14.43 ± 1.79	0.2957
SBP (mmHg)	126.4 ± 3.53	134.46 ± 4.22	0.1483
DBP (mmHg)	75.20 ± 1.86	75.57 ± 2.22	0.8985
FBG (mmol/L)	9.69 ± 0.65	8.59 ± 0.78	0.6259
HbA1c(%)	9.86 ± 0.39	9.13 ± 0.45	0.2236
TC (mmol/L)	3.98 ± 0.20	4.02 ± 0.24	0.8846
TG (mmol/L)	1.72 ± 0.16	1.45 ± 0.20	0.2913
HDL-c (mmol/L)	0.97 ± 0.04	0.90 ± 0.04	0.2208
LDL-c (mmol/L)	2.46 ± 0.15	2.59 ± 0.18	0.5831
ALT(U/L)	20.23 ± 3.75	23.60 ± 4.43	0.5625
AST(U/L)	16.38 ± 3.17	24.11 ± 3.73	0.1197
γ-GT(U/L)	25.13 ± 6.54	38.48 ± 7.86	0.1965
WBC(10^9^/L)	7.67 ± 0.68	10.15 ± 0.81	0.0220*
Neutrophil(%)	65.10 ± 2.12	70.92 ± 2.53	0.0828
Monocytes(%)	9.38 ± 1.75	14.27 ± 2.09	0.7780
Lymphocyte(%)	22.01 ± 1.46	12.57 ± 1.74	0.0001*
CRP (ng/mL)	13.95 ± 9.56	56.12 ± 10.93	0.0052*
ESR (mm/h)	15.56 ± 4.81	71.78 ± 5.56	< 0.0001*
Hypertension(n,%)	28 (70.0%)	17 (60.7%)	0.4270
Coronary heart disease(n,%)	10 (25.0%)	8 (28.6%)	0.7431
Cerebral vascular disease(n,%)	13 (32.5%)	8 (28.6%)	0.7294
Diabetic retinopathy(n,%)	20 (50.0%)	10 (35.7%)	0.2429
Diabetic nephropathy(n,%)	15 (37.5%)	7 (25.0%)	0.2782
Amputation(n,%)	0 (0.0%)	10 (35.7%)	–

*SBP* Systolic blood pressure, *DBP* Diastolic blood pressure, *FBG* Fasting plasma glucose, *HbA1c* Glycated hemoglobin A1c, *TC* Total cholesterol, *TG* Triglyceride, *HDL-c* HDL cholesterol, *LDL-c* LDL cholesterol, *ALT* Alanine aminotransferase, *AST* Aspartate aminotransferase, *γ-GT* γ-glutamyl transpeptidase, *WBC* White blood cell, *CRP* C-reactive protein, *ESR* Erythrocyte sedimentation rate. * indicates the difference between groups reaching significance

### Serum ANGPTL2 levels were increased in the presence of and advanced stage of DFUs

**Fig. 1:**
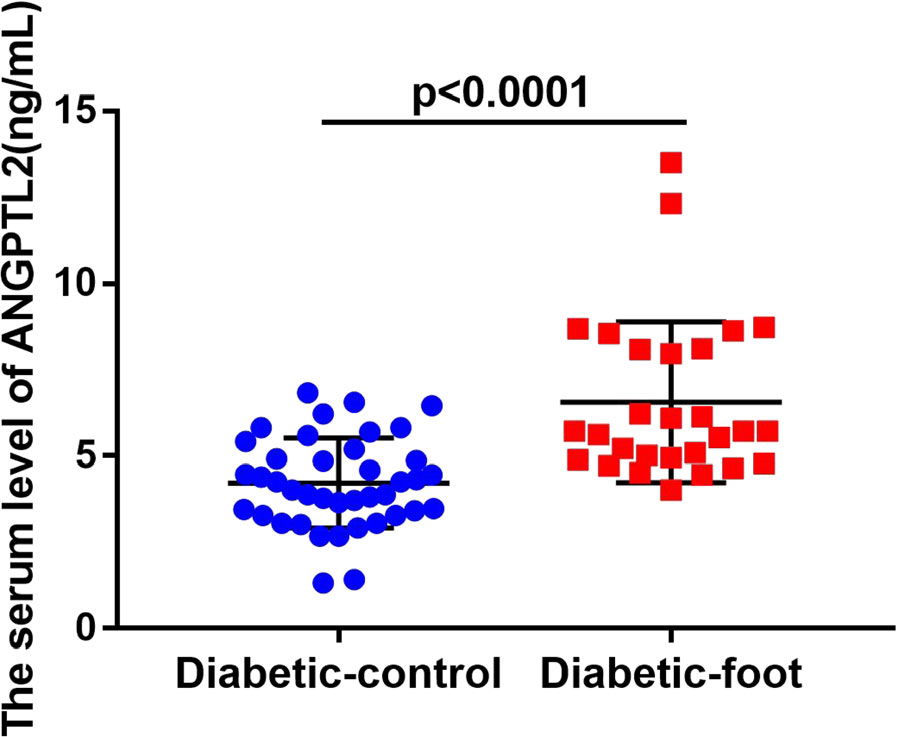
Serum ANGPTL2 levels in diabetic patients and DFU patients. Serum ANGPTL2 was significantly higher in the DFU group, when compared with the diabetic patients group. A t-TEST was used to to determine statistical significances, *p* < 0.0001

**Fig. 2:**
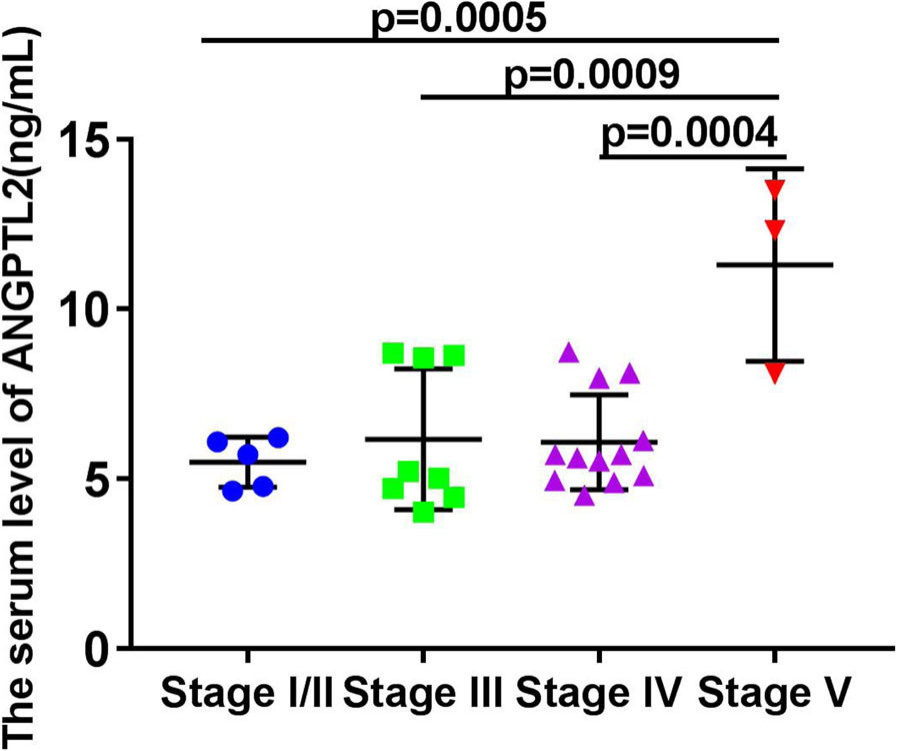
Serum ANGPTL2 levels in different DFU stages. ANGPTL2 levels increased in the advanced stage of DFU. One-way ANOVA and Turkey’s multiple comparison TEST were used to determine statistical significances, *p* = 0.0004; Stage I/II vs Stage V:*p* < 0.01; stage III vs stage V:*p* < 0.0001; stage IV vs stage V:*p* < 0.0001

Patients with DFU exhibited elevated ANGPTL2 levels when compared to subjects with T2DM only (T2DM vs. DFU: 4.221 ± 1.301 vs. 6.561 ± 2.335 μg/L, *p* < 0.0001) (Fig. 1). Next, to examine the association of ANGPTL2 with DFU severity, serum levels of ANGPTL2 in patients with DFUs at different DFU stages were compared in patients with DFU. As shown in Fig. 2, serum ANGPTL2 levels were enhanced in patients with DFUs at higher stages (5.494 ± 0.7346 vs. 6.165 ± 2.071, 6.084 ± 1.402, 11.3 ± 2.843, *p* = 0.0004, Fig. 2). Taken together, these results show that serum ANGPTL2 levels increased in the presence of and advanced stage of DFUs.

### Correlation of serum ANGPTL2 with CRP, ESR, and VEGF in all subjects

**Fig. 3:**
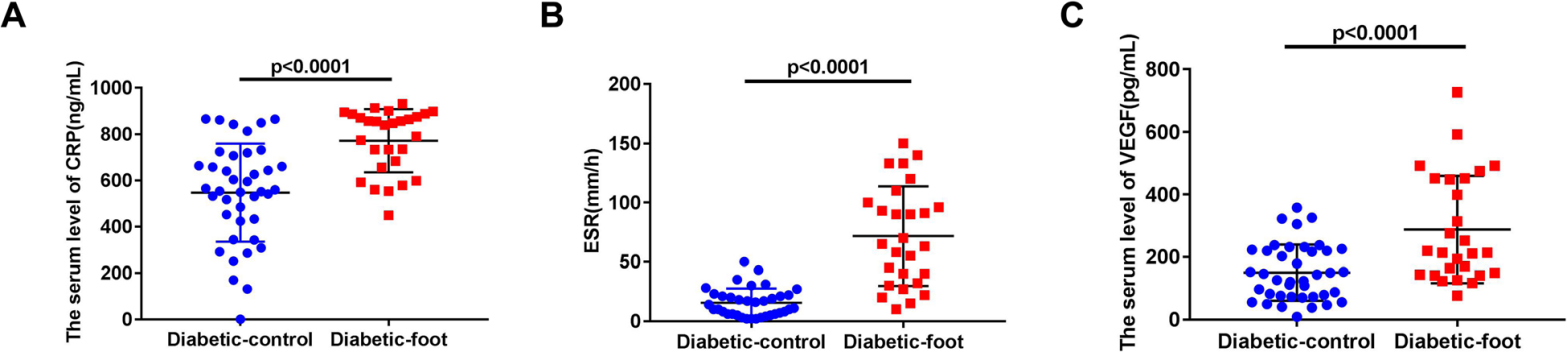
CRP, ESR, and VEGF levels in the diabetic control group and DFU group. **a**.CRP levels were increased in the presence of DFU, CRP: *p* < 0.0001. **b**. ESR levels were increased in the presence of DFU, ESR: *p* < 0.0001. **c**. VEGF levels were increased in the presence of DFU, VEGF: *p* < 0.0001. A t-TEST was used to determine statistical significances

We analyzed the levels of CRP, ESR and VEGF in both diabetic controls and patients with DFUs, and found CRP, ESR, and VEGF levels were increased in the presence of DFUs (CRP: 556.50 (426.10, 696.40) vs. 843.70 (663.4, 884.2), *p* < 0.0001, Fig. 3a; ESR: 12.50 (6.00, 21.75) vs. 65.00 (32.00, 100.00), *p* < 0.0001, Fig. 3b; VEGF: 134.80 (74.02, 222.10) vs. 214.00 (143.60, 451.50), *p* < 0.0001, Fig. 3c).

**Fig. 4:**
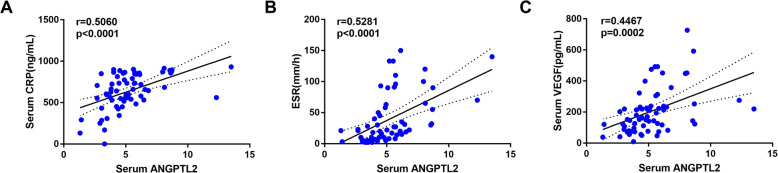
Correlations between serum levels of ANGPTL2 and CRP, ESR and VEGF levels in all subjects. **a**. Serum level of ANGPTL2 was positively correlated with CRP, *r* = 0.5060, *p* < 0.0001; **b**. Serum level ANGPTL2 was positively correlated with ESR, *r* = 0.5281, *p* < 0.0001; **c**. Serum level ANGPTL2 was positively correlated with VEGF, *r* = 0.4467, *p* = 0.0002. Spearman correlation analysis was conducted to examine the correlations

Next, the correlations between circulating ANGPTL2 levels and serum levels of CRP, ESR, and VEGF were analyzed. Spearman correlation analysis demonstrated that the serum ANGPTL2 levels were significantly positively correlated with CRP(*r* = 0.5060, *p* < 0.0001), ESR(*r* = 0.5281, *p* < 0.0001), and VEGF(*r* = 0.4467, *p* = 0.0002) levels (Fig. 4).

### Circulating ANGPTL2 levels were positively related to the stage of DFUs

Furthermore, we evaluated the associations between serum ANGPTL2 levels and various clinical parameters and the stage of DFUs (Table 2). There was a strong positive relationship between the serum ANGPTL2 level and DFU stage (*r* = 0.5914, *p* < 0.0001). Additionally, HDL-C (*r* = − 0.3411, *p* = 0.0052) and monocytes (*r* = 0. 2464, *p* = 0.0428), and lymphocytes (*r* = − 0.2915, *p* = 0.0159) were also significantly related to the serum ANGPTL2 level.

**Table 2:** Correlation between serum ANGPTL2 and clinical variables

Variables	r	p
Age	−0.1095	0.3743
Diabetes duration	0.06241	0.6131
SBP	0.1493	0.2244
DBP	0.05453	0.6587
FBG	0.1026	0.4125
HbA1c	0.05373	0.6683
TC	0.04353	0.7285
TG	0.1225	0.3272
HDL-c	−0.3411	0.0051*
LDL-c	0.1248	0.3179
ALT	0.02386	0.8480
AST	0.03918	0.7529
γ-GT	0.1009	0.4201
WBC	0.1646	0.1798
Neutrophil	0.06617	0.5919
Monocytes	0.2464	0.0428*
Lymphocyte	−0.2915	0.0159*
DFU stage	0.5914	< 0.0001*

* indicates the difference between groups reaching significance

To further estimate the correlation between serum ANGPTL2 level and DFU severity stage, we constructed a multiple linear regression model considering the serum ANGPTL2 level as the response variable and including HDL-C, monocytes, lymphocytes and DFU stage as explanatory variables, as shown in Table 2. We found that the DFU stages were still associated with the serum ANGPTL2 levels after adjustment for the risk factors (Table. 3). For this model, collinearity was assessed by calculating VIF of the models, and there was no VIF > 5**.**

**Table 3:** Multivariate model for serum ANGPTL2 and clinical variables

	β	P	95% CI	VIF
HDL-c	−1.961	0.024	−3.649- -0.273	1.099
Monocytes	0.042	0.018	0.008–0.076	1.064
**DFU stage**	0.531	< 0.001	0.301–0.760	1.139

According to Table 2, the stepwise multiple regression model was adjusted for HDL-c, Monocytes and Lymphocyte. Estimates β with 95% confidence intervals (CI) were shown for the model

### ANGPTL2 was demonstrated to be an independent risk factor for DFUs

To elucidate the independent effect of the serum ANGPTL2 level on the presence of DFU, binary logistic regression analysis was conducted (Table 4). In the model, after adjustment for the age, sex, history of T2DM, and HbA1c, which are common risk factors for diseases, the data suggested that ANGPTL2 was an independent risk factor for DFU (β = 1.2759, OR (95% CI): 3.58(1.81–7.10), *p* = 0.0003).

**Table 4:** ANGPTL2 was demonstrated to be an independent risk factor for diabetic foot

	β	S.E	OR(95% CI)	p
Sex	−0.4895	0.3616	0.96 (0.92–1.01)	0.1758
Age	−0.0221	0.0258	1.07 (0.97–1.08)	0.3915
T2DM course	−0.0077	0.0373	1.01 (0.94–1.08)	0.8357
HbA1c	−0.3548	0.1887	0.70 (0.48–1.02)	0.0601
ANGPTL2	1.2759	0.3493	3.58 (1.81–7.10)	0.0003*

* indicates the difference between groups reaching significance

## Discussion

In this study, 68 T2DM participants were recruited, including 28 with DFU and 40 without DFU. The blood biochemical criteria and ANGPTL2 and VEGF levels were measured. The key findings of this study are summarized as follows: (1) serum ANGPTL2 levels were higher in the DFU group compared with the control (T2DM); (2) serum ANGPTL2 levels were higher in the advanced stages of DFU; (3) serum CRP, ESR, and VEGF levels were increased in the presence of DFUs; (4) serum ANGPTL2 levels were significantly positively related to CRP, ESR, and VEGF levels, individually; (5) serum ANGPTL2 was positively correlated with diabetic foot stage after adjusting the risk factors; (6) ANGPTL2 was demonstrated to be an independent risk factor for DFUs. Therefore, serum ANGPTL2 was considered to play important roles in the development of DFU.

Vascular endothelial cell injury in the lower extremities is an important factor in the formation of DFU [18]. Under normal physiological conditions, the injured vascular endothelial cells are repaired by the surrounding endothelial cells and endothelial precursor cells. However, high glucose levels could stimulate endothelial cells to increase the transcription activity of NF-κB [19], resulting in the invasion of peripheral inflammatory cells, the production of inflammatory mediators and the production of peroxides, which decrease the compensatory regeneration ability of endothelial cells. ANGPTL2 is a proinflammatory factors. When secreted by perivascular adipose tissue around blood vessels, it accelerated the development of vascular inflammation and the subsequent neointimal hyperplasia after endothelial injury in mice [20]. In addition to acting as an inflammatory mediator, ANGPTL2 is also related to vasodilation. The expression of eNOS in ApoE(−/−)/Tie2-Angptl2 Tg mice decreased, leading to impaired NO-mediated vasodilation [21]. Angptl2(−/−) mice exhibited less severe endothelial dysfunction compared with wild-type mice when fed a high-fat diet [21]. Furthermore, ANGPTL2 has been reported to be involved in the progression of cardiovascular diseases. It was highly expressed in vascular endothelial cells and macrophages in the atherosclerotic vessels of patients with coronary heart disease [21]. The Hisayama Study suggested that elevated serum ANGPTL2 levels are a novel risk factor for the development of CVD in the Japanese general population [22]. Serum ANGPTL2 is also a new candidate biomarker for risk stratification of acute coronary syndrome [23]. In addition, clinical studies have reported that the ANGPTL2 level is a risk factor for lower extremity artery disease and lower extremity amputation in a large cohort of T2DM patients [15, 16], while peripheral arterial diseases and amputation were the ultimate consequences of DFUs. These results suggested that the serum ANGPTL2 level might be associated with DFU development. Therefore, this study aimed to explore the potential connection between ANGPTL2 and DFUs.

We found that serum ANGPTL2 levels were increased in the DFU group compared with the T2DM control group, and the ANGPTL2 levels were increased as the severity of the DFU increased. These results are consistent with previous studies showing that serum ANGPTL2 levels were high in patients with chronic diseases, such as heart disease [13], kidney disease [24], obesity [11], diabetes [12] and cancer [25]. Several studies have shown that ANGPTL2 exhibited both physiological and pathological functions [10]. Although some studies have reported that ANGPTL2 promoted pro-angiogenic activity, anti-apoptotic activity and endothelial cell migration, as do the ANGPTLs, the increased levels of ANGPTL2 in patients with a DFU may be due to a compensatory increase. In pathological conditions, endothelial cells and infiltrated macrophages producing excess ANGPTL2 induced an inflammatory response by activating NF- κB signaling, which promoted endothelial dysfunction and atherosclerosis progression. And endothelial dysfunction and lower limb atherosclerosis were two key factors in the occurrence and development of DFU. However, whether ANGPTL2 was involved in the occurrence and development of DFU and whether blocking ANGPTL2 could delay the occurrence and development of DFU requires further study.

CRP and ESR were demonstrated to be important classical biomarkers for increasing risk of DFU. VEGF, a growth factor, is a key regulator of integrin-mediated vascular pathways, which participated in the wound healing and played an important role in the development of DFUs. VEGF facilitated vascular endothelial migration, proliferation, and capillary-like network formation in vitro, and promoted vasculogenesis, angiogenesis and vascular permeability in vivo [26]. In the present study, we found VEGF levels increased in DFU, which might result from a compensatory increase in VEGF under pathological conditions. Moreover, CRP and ESR levels were increased in the presence of DFU, and CRP, ESR, and VEGF were positively and significantly related to serum ANGPTL2 levels, suggesting that ANGPTL2 exactly acted as a risk factor for DFUs. Age, diabetes duration, smoking, drinking, poor glucose control, hypertension and hyperlipidemia are the traditional risk factors for DFU in diabetic patients. However, serum ANGPTL2 levels displayed poor correlations with these factors, which may be due to the small number of subjects or other factors affecting the level of serum ANGPTL2. Correlation analyses and multivariate linear regression analyses confirmed that serum ANGPTL2 was positively correlated with the DFU stage. Binary logistic regression analysis demonstrated ANGPTL2 was an independent risk factor for diabetic foot after adjusting for age, gender, HA1C, and duration of diabetes.

However, there are some limitations in this study. First, the small sample size might decrease the statistical power of our results. Due to the strict criteria for inclusion, the study comprised 68 individuals, including 40 diabetic control and 28 DFU patients, and we will further expand the sample size to verify the results of the study in the future. In addition, we found that serum ANGPTL2 levels were an independent risk factor for DFU, but further investigation is needed to uncover the effect of ANGPTL2 on DFU and its mechanism of action. Lastly, the present study was a cross-sectional study and longitudinal studies are needed to determine whether ANGPTL2 levels predict the incidence and severity of DFUs.

## Conclusions

In conclusion, circulating ANGPTL2 levels are an independent risk factor for DFUs and are closely related to the severity of diabetic foot. ANGPTL2 may play important roles in the development of DFUs, but this need to be investigated in prospective studies.

## Data Availability

The data used and/or analyzed during the current study are available from the corresponding author on reasonable request.

## Abbreviations

ANGPTL2: Angiopoietin-like 2
T2DM: Type 2 diabetes mellitus
DFU: Diabetic foot ulcers
SBP: Systolic blood pressure
DBP: Diastolic blood pressure
FBG: Fasting plasma glucose
OGTT: Oral glucose tolerance test
HbA1c: Glycated hemoglobin A1c
TC: Total cholesterol
TG: Triglyceride
HDL-c: HDL cholesterol
LDL-c: LDL cholesterol
ALT: Alanine aminotransferase
AST: Aspartate aminotransferase
γ-GT: γ-glutamyl transpeptidase
WBC: White blood cell
CRP: C-reactive protein
ESR: Erythrocyte sedimentation rate
VEGF: Vascular endothelial growth factor
VIF: Variance inflation factor
